# Camelid Inoculation with Middle East Respiratory Syndrome Coronavirus: Experimental Models of Reservoir Host Infection

**DOI:** 10.3390/v12121370

**Published:** 2020-11-30

**Authors:** Danielle R. Adney, Chad S. Clancy, Richard A. Bowen, Vincent J. Munster

**Affiliations:** 1 Laboratory of Virology, Division of Intramural Research, National Institute of Allergy and Infectious Diseases, National Institutes of Health, Hamilton, MT 59840, USA; danielle.adney@nih.gov; 2 Rocky Mountain Veterinary Branch, National Institute of Allergy and Infectious Diseases, Hamilton, MT 59840, USA; chad.clancy@nih.gov; 3 Department of Biomedical Sciences, Colorado State University, Fort Collins, CO 80521, USA; rbowen@rams.colostate.edu

**Keywords:** MERS-CoV, camelid, coronavirus pathogenesis, animal coronaviruses

## Abstract

Within the past two decades, three zoonotic betacoronaviruses have been associated with outbreaks causing severe respiratory disease in humans. Of these, Middle East respiratory s yndrome coronavirus (MERS-CoV) is the only zoonotic coronavirus that is known to consistently result in frequent zoonotic spillover events from the proximate reservoir host—the dromedary camel. A comprehensive understanding of infection in dromedaries is critical to informing public health recommendations and implementing intervention strategies to mitigate spillover events. Experimental models of reservoir disease are absolutely critical in understanding the pathogenesis and transmission, and are key to testing potential dromedary vaccines against MERS-CoV. In this review, we describe experimental infections of dromedary camels as well as additional camelid models used to further understand the camel’s role in MERS-CoV spillover to humans.

## 1. Introduction

The Middle East respiratory syndrome coronavirus (MERS-CoV) is a zoonotic coronavirus originally isolated from a man with fatal acute pneumonia that progressed to renal failure [1]. MERS-CoV joins the severe acute respiratory syndrome virus (SARS-CoV) and SARS-CoV-2 as one of three newly emerging, highly pathogenic betacoronavirus infecting humans. Since its discovery in 2012, there have been just over 2500 confirmed human MERS cases, with a case fatality rate of 34% [2]. Humans infected with MERS-CoV can present with a variety of non-specific respiratory symptoms ranging from an asymptomatic infection to severe disease with acute respiratory distress syndrome (ARDS) and multiorgan failure. As seen with Coronavirus Disease 2019 (COVID-19) patients, MERS patients with co-morbidities are at an increased risk of severe disease [3,4].

The identification of the reservoir host for emerging infectious disease is a critical step in controlling outbreaks. Bats were initially investigated as a potential reservoir, as phylogenetically the virus resembled two additional betacoronaviruses isolated from bats—BtCoV-HKU4 and BtCoV-HKU5 [5]. Interestingly, bats were also suggested to play a role in the 2002–2003 (SARS-CoV) outbreak [6,7]. A 2012 field surveillance study of *Taphozous perforates* bats captured near an index case-patient in Bisha, Saudi Arabia, yielded a fecal sample containing virus with 100% nucleotide homology to a human MERS-CoV isolate [8]. However, several patients also reported contact with sick or pyrexic goats and camels prior to infection [9,10,11]. These early reports prompted a wider search for potential proximate reservoir hosts. Focus quickly shifted to dromedary camels after an initial report of nearly uniform seropositivity in sampled dromedaries [12].

While contact with dromedary camels has not been identified in every human case, multiple instances of camel-to-human spillover have been described. In 2013, samples collected from a fatal human case and the patient’s nine camels suggested recent infection within the herd [13]. Genomic sequencing from one of the camels and the patient revealed identical sequences, strongly suggesting an instance of zoonotic transmission [13]. Interestingly, prior to becoming ill, the patient reported nasal discharge in several of his camels and described administering a topical medication and therefore had direct contact with camel nasal secretions [13]. Active screening of dromedaries imported into United Arab Emirates and tracing of subsequent human contacts identified multiple asymptomatic human infections [14]. Approximately 50% of camel workers tested in Saudi Arabia had detectable antibodies against MERS-CoV as compared to less than 4% of the general population in the country [15,16]. An important step in decreasing the number of human cases relies on identifying risk factors associated with handling camels and taking actions to mitigate these risks.

Early serosurveillance of dromedary camels indicated nearly uniform exposure to MERS-CoV, whereas MERS-CoV specific antibodies were not readily detectable in other livestock such as small ruminants, cattle, and equids [12,17,18,19]. A high proportion of camels in Africa, the Middle East, and some camels in the Canary Islands are seropositive; however, camels sampled in Australia were seronegative [12,20]. Both viral RNA and infectious virus are detectable in nasal swabs collected from actively infected dromedaries, with a higher prevalence of viral shedding in juvenile animals and a higher prevalence of seropositivity in mature animals [21,22,23]. Interestingly, neutralizing antibodies and viral RNA have been detected in other secretions, including milk. However, because calves nurse prior to milking, viral contamination from infected calves could likely explain the presence of viral RNA in milk. Virus assays from experimentally inoculated camel milk indicated that virus was stable for several days, highlighting the possibility of transmission through raw milk consumption [24,25]. This widespread transmission amongst camels underscores the importance of a comprehensive understanding of pathogenesis, shedding, and transmission of camels infected with MERS-CoV.

Additional camelid species have been evaluated for natural exposure to MERS-CoV. Unlike dromedary camels, Bactrian camels and New World camelids (NWCs) may not be naturally exposed to or become infected by the virus at the same frequency due to natural geographic separation. Serosurveillance studies of Bactrian camels in Mongolia, Kazakhstan, and the Netherlands failed to identify animals shedding virus or animals positive by serology. However, seropositive Bactrian camels were identified in Dubai, suggesting a unique geographic distribution of MERS-CoV [26,27,28,29]. Serologic testing of a mixed herd of dromedaries and alpacas in Qatar indicated that all alpacas had MERS-CoV specific antibodies; albeit with titers lower than the sampled dromedaries in the herd [30]. Nasal swabs from both camels and alpacas in this herd were negative for viral RNA [30]. Sampling from camelids in Israel between 2012 and 2017 did not identify any actively infected animals through RT-PCR of nasal swabs; however, seropositive camels, alpaca, and llamas were identified [31].

## 2. Camelid Taxonomy and General Biology

The Camelidae family comprises three genera: *Camelus*, *Lama*, and *Vicugna*. The genus *Camelus* contains the Old World camelids (OWC) and includes the dromedary camel (*C. dromedarius*) and the Bactrian camel, which is comprised of two genetically distinct species: wild Bactrian camels (*C. bactrianus ferus*), and domesticated Bactrian camels (*C. bactrianus bactrianus*) [32]. NWCs or South American camelids (SAC) include the genera *Lama* and *Vicugna*, both containing a wild (*L. guanaco* and *V. vicugna*) and domesticated (*L. glama* or llama and *V. pacos* or alpaca) species [33]. Like other mammals, camelids produce double-chained antibodies. However, camelids are unique in that they also produce heavy-chain antibodies that do not have a light chain known as single chain antibodies or nanobodies [34,35]. These camelid nanobodies have shown to be useful therapeutically and inspired a new class of therapeutics that has resulted in drugs such as Caplacizumab [36]. These heavy chain antibodies have also been adapted to as a candidate therapeutic that showed promise in transgenic mice infected with MERS-CoV [37].

The camelid respiratory system has several unique features and differs slightly between OWC and SAC. Unlike SAC, camels are capable of closing their nostrils completely to prohibit dust from entering the respiratory tract. Camelid turbinates are delicate and anatomically resemble those of cattle or sheep. Similar to equids, camelid lungs do not have true lobation with the exception of an accessory lobe on their right lung around the caudal vena cava. Camelid erythrocytes are ellipsoid in shape for greater surface area and thus, oxygen carrying capacity. SACs are uniquely adapted to survival in high attitudes by maintaining a polycythemia, hemoglobin with a higher affinity for oxygen, and an oxygen dissociation curve that is shifted to the left [38,39]. Interestingly, SACs are able to maintain a mild to moderate pulmonary hypertension without pathologic remodeling of the pulmonary artery or right heart. In contrast, cattle raised at high-altitude frequently develop a fatal pulmonary hypertension or elevated pulmonary arterial pressure known as high-altitude disease [40]. While these differences likely do not account for camelids’ mild disease during MERS-CoV infection, the ability for camelids to be clinically asymptomatic in low oxygen tension environments is important to consider when evaluating the clinical progression of MERS-CoV in animal models.

## 3. Camelid Study Design

Experimental infections of camelids with MERS-CoV require specialized bio-containment facilities due to these animals’ size and temperament. To date, only three high-containment facilities have successfully experimentally infected camelids with MERS-CoV: the Commonwealth Scientific and Industrial Research Organisation (CSIRO) Australian Animal Health Laboratory, the Animal Disease Laboratory at Colorado State University (CSU), and the Centre de Reeceerca en Sanitat Animal (CReSA) in Spain [41,42,43,44,45,46,47,48]. The groups at CReSA and CSU performed camelid transmission experiments that involved co-housing experimentally inoculated animals with naïve animals to study MERS-CoV transmission dynamics [44,46]. Additionally, Alharbi et al. reported a novel facility and challenge design in which naïve animals were infected through contact with naturally infected camels in outdoor pens to model infection in camel herds [49,50].

## 4. Clinical Signs

Experimental infections indicate that both OWC and SAC camelids are susceptible to MERS-CoV infection. Infection is characterized by minor clinical signs comprised of mild to moderate nasal discharge in dromedary camels, Bactrian camels, and llamas [42,43,45,46,47,49,50]. Nasal discharge in alpacas has not been reported [41,44]. One study reported a reduced condition score in a single alpaca 18 days post-infection (DPI); however, at necropsy, this animal had extensive adhesions associated with the first stomach compartment (C1) to the umbilicus and the reduced condition was likely a result of this lesion rather than MERS-CoV infection [41] (Table 1).

## 5. Viral Shedding

MERS-CoV has predominantly been detected in dromedary nasal swabs; thus, experimental infections in camelids have focused exclusively on respiratory inoculation. The majority of studies utilized an intranasal inoculation route with 10^7^ plaque-forming units (PFU) or median tissue culture infectious dose (TCID_50_) by direct intranasal exposure, nebulization, or a mucosal atomization device [46,47]. Crameri et al. inoculated three alpacas oronasally with 10^6^ TCID_50_ MERS-CoV [41]. Other studies challenged animals by direct contact with infected animals in order to study transmission or natural infection in camelids [44,46,49,50].

During infection, MERS-CoV is primarily shed in nasal secretions of infected camelids. Adney et al. reported detectible infectious virus in dromedary camels beginning on 1 DPI and continuing until 7 DPI, with viral RNA detectable for up to 35 days [45] (Table 1). Similarly, Haagmans et al. detected infectious virus in inoculated camels for 6 days post-inoculation with detectible RNA until euthanasia at 14 DPI [47]. Two experimentally infected Bactrian camels shed infectious virus beginning on 1 DPI and continued until euthanasia on 5 DPI or 7 DPI [42]. While low levels of infectious virus and viral RNA can be detected in oral swabs, it is believed to be the result of drainage from the respiratory tract rather than replication in the oral cavity [45]. Shedding through feces does not appear to contribute to transmission, as one study only detected low levels of viral RNA in rectal swabs and Adney et al. did not detect infectious virus or RNA in fecal samples [45,47]. Infectious virus or viral RNA has not been detected in urine, whole blood, or serum [45,47].

As with OWCs, studies with NWCs also suggest that viral shedding is primarily constrained to nasal secretions. Infectious virus in nasal swabs was detected in seven of eight llamas, although virus was not isolated from every animal on every day, and infectious virus was not detected after 7 DPI. Viral RNA was detected from all animals and continued in one of four remaining animals until 15 DPI [48]. Alpacas infected intranasally with 10^7^ plaque-forming units (PFU) MERS-CoV began shedding at 1 DPI, with infectious virus detected in one of three animals at 7 DPI [44]. In contrast, three alpacas infected oronasally with 10^6^ TCID_50_ MERS-CoV had intermittently detectable infectious virus in oral and nasal swabs through day 12, although the infectious virus was not quantified [41]. This latter study reported that viral RNA was detected in oral and nasal swabs from 3–12 DPI [41]. Viral shedding from NWCs appears to be less commmonly detected compared to experimentally-infected OWC (Table 1).

## 6. MERS-CoV Pathologic Lesions

**Figure 1:**
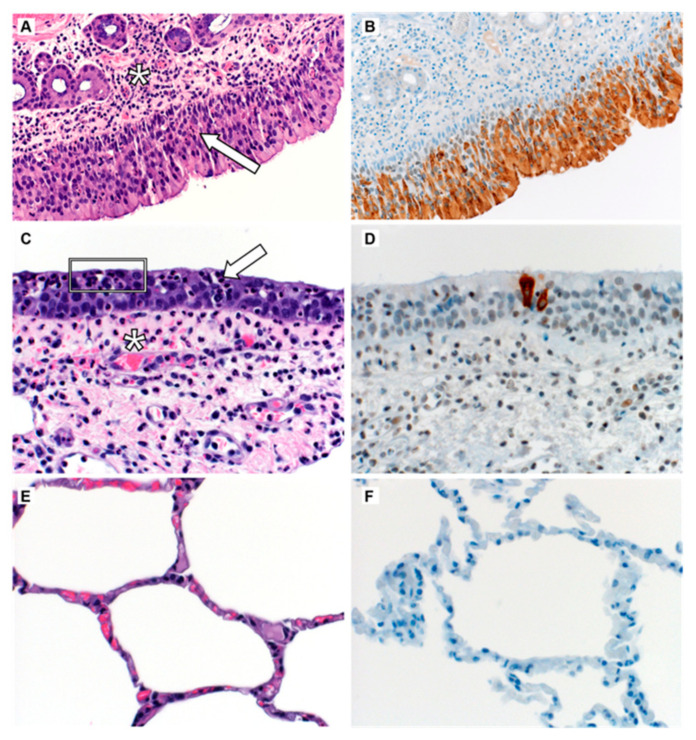
Histopathology and Immunohistochemistry of a Bactrian Camel Experimentally Inoculated with Middle East Respiratory Syndrome Coronavirus (MERS-CoV). (**A**) Within the nasal turbinates, the lamina propria (asterisk) and mucosa (arrow) are infiltrated by moderate numbers of neutrophils with fewer lymphocytes and macrophages. (**B**) The neurosensory ciliated epithelium of the nasal turbinates show strong immunoreactivity to MERS-CoV antigen. (**C**) Within the trachea, there is loss of cilia (ciliocytophthoria, white box) and infiltration of the mucosa (arrow) and lamina propria (asterisk) by moderate numbers of neutrophils. (**D**) There is limited immunoreactivity of ciliated epithelial cells to MERS-CoV antigen. (**E**) No histopathologic lesions are noted in the lower respiratory tract. (**F**) Immunoreactivity to MERS-CoV antigen is not detected in the lung. Magnification: 400×.

The respiratory tract of mammals can be divided into two distinct anatomic regions: the upper respiratory tract, and the lower respiratory tract. One distinct histologic difference between these two regions is the presence of ciliated epithelium lining the bulk of the upper respiratory tract, while the lower respiratory tract is lined primarily by cuboidal or squamous epithelium. In the acute phase of disease in experimental MERS-CoV infections, the primary histopathologic lesions are limited to the upper respiratory tract (Figure 1). Consistently, MERS-CoV inoculation has been shown to result in epithelial cell necrosis, mucosal ulceration, increased mucous production and neutrophil accumulation in the nasal mucosa [42,45,48,51]. Common to other upper respiratory viral infections, lymphocytic infiltration and aggregation has been consistently noted in the upper respiratory submucosa of experimentally inoculated camelids [48]. Lower in the respiratory tract during the acute phase of infection, the inflammatory process is limited to epithelial necrosis and lymphocytic infiltration and squamous metaplasia during the acute phase of disease [51]. Consistent with the histopathology, viral antigen has been primarily detected in the upper respiratory tract of camelids with few reports of viral antigen present lower in the respiratory tree [42,45,48,51] (Figure 1). To date, viral antigen has not been detected within the terminal airways or at the level of the alveolus.

One subtle lesion noted both histologically and ultrastructurally in both New and Old World camelids is ciliary loss (ciliocytophthoria) [45,51]. Ciliocytophthoria is less pronounced in New World camelids [48]. This lesion is non-specific and has been previously documented in other upper respiratory viral infections such as respiratory syncytial virus in humans [52]. Lack of ciliary activity in the respiratory tract hinders removal of cellular debris, inhaled foreign material and bacteria. Theoretically, loss of cilia would predispose the respiratory tract to secondary bacterial infection. Identifying the mechanisms by which cilia presence and function are lost and subsequently regained may be a key focus of future scientific investigations on upper respiratory viral infections.

MERS-CoV lesions outside of the upper respiratory tract have been noted to be mild and limited in experimental infections. Lymph nodes and lymphoid tissue that drains the respiratory tract (tonsil, retropharyngeal, submandibular and mediastinal lymph nodes) have been evaluated in several studies. The changes observed on histologic evaluation are non-specific and are consistent with a reactive immune response including lymphoid follicular hyperplasia and individualized apoptotic lymphocytes [51]. No additional significant histologic lesions have been reported to date outside of the respiratory tract in any camelid model of MERS-CoV.

## 7. Transmission of MERS-CoV amongst Camelids

The high seroprevalence reported in dromedary herds suggests efficient camel-to-camel transmission of virus. Animal-to-animal transmission has been demonstrated in dromedary camels, llamas, and alpacas. Alharbi et al. co-housed naïve and vaccinated camels with naturally infected camels, demonstrating MERS-CoV transmission [49,50]. Viral RNA was detected in nasal swabs from the naïve dromedary calves after exposure to naturally infected calves within 24 to 48 h [49]. Surprisingly, control calves housed 200 m away from infected animals seroconverted over the course of one study, indicating that transmission is likely extremely efficient [49,50]. Interestingly, multiple studies report that not all co-housed NWC shed detectible infectious virus indicating that transmission may be less efficient than with OWC [44,46]. Naïve co-housed llamas seroconverted and shed viral RNA, although infectious virus was only isolated from three of five naïve llamas [46]. Similarly, all co-housed naïve alpacas seroconverted and infectious virus was detected in only two of three animals [44]. Given the high prevalence of seropositive dromedaries in the Middle East and Africa, this efficient animal-to-animal transmission is not surprising and highlights the importance of limiting camel infection to lower human exposure.

There has been considerable interest concerning the similarity between experimentally and naturally infected camelids given the high inoculation doses used. In-contact animals have been used as a proxy for naturally infected animals to better understand transmission and shedding dynamics in camelids. In all studies, animals infected by contact had similar clinical signs to experimentally infected animals, although clinical signs alone appear to have a low sensitivity for detecting active infection [44,47,49]. Similarly, co-housed animals with detectable infectious virus or viral RNA generally shed levels that approximate the doses given to experimentally infected animals. Finally, while some co-housed animals have lower serological titers than experimentally infected animals, co-housed camelids readily seroconvert after exposure to infected animals.

DPP4′s binding affinity to the viral spike (S) protein as well as tissue distribution likely play an important role in viral transmission. In vitro analysis indicates that camel, human, and equid DPP4 strongly binds S protein while DPP4 in other hoofstock as cattle and small ruminants binds less efficiently [53,54]. DPP4 is readily expressed in the upper respiratory tract of dromedary camels while humans express DPP4 primarily in the lower respiratory tract [55]. This distribution difference could potentially explain the difference in transmission efficiency between dromedaries and humans.

## 8. MERS-CoV Vaccination

Although MERS-CoV-infected camels do not suffer from overt clinical disease, vaccination and subsequent herd immunity could limit viral circulation between camel populations and limit human exposure. Several reinfection studies of alpacas suggest that previous infection can protect against reinfection at 21 DPI and 70 DPI, although the duration of this protection is unknown [41,44]. Several field studies indicate a possibility of re-infection in dromedaries, making it difficult to protect against continued exposures over the course of an animal’s life [56,57]. Like several human vaccine candidates, current camelid vaccine trials have utilized viral vector-based vaccines expressing S protein as well as recombinant S protein vaccines in order to target the glycoprotein responsible for cell binding [43,46,47,49].

Viral vector-based vaccines are an attractive vaccine platform due to their ability to induce both humoral and cellular immunity [58]. Modified vaccinia virus Ankara (MVA-s) and chimpanzee adenoviral vectors have been used in camels to induce immunity against the spike protein and reduce viral shedding [47,49]. Camels were vaccinated with the MVA-s vaccine intramuscularly and intranasally with a mucosal atomization device, and boosted 4 weeks later, resulting in induction of neutralizing antibodies in all vaccinated animals [47]. After exposure to the virus, vaccinated animals had significantly decreased viral RNA and infectious virus in nasal swabs as compared to control animals [47]. Vaccinated animals also produced neutralizing antibodies to camelpox virus, a pathogen of veterinary importance associated in decreased production, high morbidity, and high mortality in camels [59,60].

An additional vaccine platform evaluated in dromedaries is ChAdOx2 MERS, a chimpanzee adenovirus-based vaccine platform that has shown promise in primates for MERS-CoV and that is currently in human clinical trials for use against SARS-CoV-2 [49,61,62]. Both seronegative and seropositive animals were vaccinated, resulting in the development of antibodies in naïve animals and an increase in titer in previously seropositive animals [49]. Importantly, previously seronegative animals under 1 year of age required two doses of ChAdOx2 MERS, while previously seronegative animals over 1 year of age only required a single dose to induce an immune response [49]. After exposure to MERS-CoV, previously seropositive vaccinated animals shed the least amount of detectable viral RNA, followed by previously seropositive control animals [49]. In contrast, animals that were previously seronegative (in both the control and vaccinated groups) shed the most viral RNA after exposure to virus [49].

When vaccinated with an adjuvated MERS-CoV spike protein subunit vaccine, two of two alpacas and two of three camels developed neutralizing antibodies against the virus [43]. While this vaccine resulted in similar humoral responses between species, alpacas were completely protected against experimental infection as compared to reduced and delayed shedding in dromedaries [43]. As transmission between camels is likely extremely efficient, this particular vaccine would likely not result in a significant decrease in camel-to-camel transmission. However, by decreasing the amount of detectable infectious virus, it could decrease the risk to camel workers by lowering their exposure to virus. Similarly, an adjuvanted recombinant spike S1-protein vaccine delivered to llamas resulted in neutralizing antibodies when delivered intramuscularly [46]. These animals were challenged through contact with experimentally infected llamas with low levels of viral RNA detected in four of five vaccinated animals and higher levels detected in the 5th vaccinated animal [46]. Interestingly, although RNA was detectable at levels similar to naïve llamas in one vaccinated animal viral, infectious virus was not isolated, indicating that mucosal neutralizing antibodies could be important in controlling infectious viral shedding [46].

## 9. Conclusions

Dromedary camels continue play an important role in the continued transmission of MERS-CoV. Experimentally infected camelids consistently develop minor clinical disease, shed virus primarily through nasal secretions, quickly clear their infection, and develop a measurable humoral response post-exposure. Adopting a ‘One Health’ strategy to reduce viral shedding in camels could be an excellent method for decreasing the frequency of spillover events to humans. Due to the fairly ubiquitous nature of the virus in Middle Eastern camelid populations, timing will play an important role in the success of any vaccination campaign. Passive immunity through colostrum likely affords protection very early in life. Similar to vaccination strategies in companion animal species, frequent vaccination may be required in juvenile animals to capitalize on the window between the loss of maternal antibodies and natural exposure to the virus. Further studies to develop candidate vaccines that effectively neutralize virus in the nasal turbinates and induce long-lasting humoral immunity will be critical to the success of any new vaccine strategy. It is still poorly understood whether natural infection affords immunity, or whether camels can be re-infected at multiple points over their lifetime. Thus, routine vaccinations may be required with boosters prior to ‘higher-risk’ activities such as transportation or mixing of animals for meaningful protection. Unlike other livestock diseases, clinical disease in infected dromedaries is either undetectable or very mild, meaning infection likely does not impact production values for commercial animals, which likely reduces the incentive to vaccinate.

One limiting factor in the advancement of vaccination campaigns is the ability to conduct well-controlled experimental trials to demonstrate efficacy. Multiple large animal ABSL3 facilities have successfully challenged camelids with MERS-CoV; however, these studies come with significant financial cost, safety risk and require specialized facilities and personnel [63]. Alpacas are significantly smaller and safer to handle as compared to camels; however, they have shown differences in vaccine efficacy as compared to dromedaries [43]. Exposing naïve dromedary camels in outdoor pens is an attractive alternative but is accompanied with several important limitations. The high seroprevalence in endemic areas can make it very difficult to acquire large numbers of naïve animals and the transmission study designs hinge on the ability to locate actively infected animals [49,50]. Unlike experimentally inoculated animals, animals infected through contact do not receive a standardized challenged dose and there is no way to control the isolate used for infection.

Understanding the viral ecology of MERS-CoV in camelids and spillover events in humans is crucial in curbing viral spillover. While dromedary camels remain the natural viral reservoir, other camelids have been important tools to study transmission and shedding dynamics. Both Old and New World camelids are capable of productive MERS-CoV infection that consists of nonspecific clinical symptoms and histopathology. Infected animals are capable of efficient viral transmission, which could potentially be mitigated through vaccination strategies. In conclusion, while these experimental infections have their unique challenges, these models are an essential branch of MERS-CoV research.

## Figures and Tables

**Table 1:** Camelid Models of MERS-CoV.

	Old World Camelids		New World Camelids	
	Dromedary Camels	Bactrian Camels	Llamas	Alpacas
Relative difficulty housing/handling	+++	+++	++	+
Nasal discharge	Present [43,45,47,49,50]	Present [42]	Present [48]	Absent [41,43,44]
Nasal shedding: infectious virus	1–6 or 7 DPI [43,47]	1–7 DPI [42]	1–7 or 8 DPI [46,48]	1–7 to 12 DPI [41,44]
Nasal shedding: viral RNA	1–35 DPI [41]	Not done	1–15 DPI [48]	1–12 DPI [41]
Pathologic lesions	Mild/moderate [42,43,47]	Mild/moderate [42]	Mild/moderate [48]	Minimal/mild [43,44]
Transmission	Present [49,50]	Not done	Present [46]	Present [44]
Vaccines tested	Recombinant S Protein [43] MVA-s [47] ChAdOx2 MERS [49]	Not done	Recombinant S Protein [46]	Recombinant S protein [43]
Vaccine efficacy	Mixed [43,47,49]	Not done	Infectious virus not detected in vaccinated animals [46]	Infectious virus not detected in vaccinated animals [43]
